# Effect of incubation duration, growth temperature, and abiotic surface type on cell surface properties, adhesion and pathogenicity of biofilm-detached *Staphylococcus aureus* cells

**DOI:** 10.1186/s13568-017-0492-0

**Published:** 2017-10-24

**Authors:** Simon Oussama khelissa, Charafeddine Jama, Marwan Abdallah, Rabah Boukherroub, Christine Faille, Nour-Eddine Chihib

**Affiliations:** 1 CNRS, ENSCL, UMR 8207-UMET-PSI, Université de Lille1, Avenue Dimitri Mendeleïev, CS 90108, 59652 Villeneuve d’Ascq, France; 2 CNRS, INRA, UMR 8207-UMET-PIHM, Université de Lille1, 369 rue Jules Guesde, CS 20039, 59651 Villeneuve d’Ascq, France; 3 CNRS, IEMN, UMR 8520, Université de Lille1, Cité Scientifique, Avenue Poincaré, 59652 Villeneuve d’Ascq, France

**Keywords:** *Staphylococcus aureus*, Physiology, Planktonic cells, Biofilm-detached cells, Surface properties, Pathogenicity

## Abstract

The goal of this study was to investigate the effect of growth conditions such as the temperature (20, 30 and 37 °C), incubation duration (24 and 48 h) and surface type (stainless steel and polycarbonate) on the cell surface physicochemical properties and adhesion to abiotic surfaces of biofilm-detached and planktonic *Staphylococcus aureus* cells. This study tested also the hypothesis that *S. aureus* planktonic cells exhibit distinct pathogenic properties compared with their sessile counterparts. The results showed that the changes of the growth conditions promoted changes in the zeta potential, hydrophobicity, electron donor/acceptor character of the studied cell populations. Biofilm-detached cells showed a greater adhesion to stainless steel and polycarbonate compared with planktonic cells. Compared with planktonic cells, sessile ones showed higher cytotoxic effect against HeLa cells, DNase activity, and siderophore levels. The higher cytotoxic effect and production of DNase and siderophore increased with the increase of temperature and duration of incubations. Based on the obtained data, the *S. aureus* biofilm-detached cells were found to be distinct in many physiological properties compared with their planktonic counterparts.

## Introduction

*Staphylococcus aureus* is an important Gram-positive human pathogen frequently associated with numerous forms of human infections (Harris et al. 2002; Khelissa et al. 2017; Valaperta et al. 2010). *S. aureus* represents the main cause of hospital acquired infections such as infections associated with indwelling medical devices and surgical wounds (Percival et al. 2015). The pathogenesis of such bacterium correlates with several virulence factors including hemotoxins, pore forming toxins, super antigens (e.g. toxic shock syndrome toxin-1, staphylococcal enterotoxin) and several secreted enzymes that result in tissue destruction and bacterial dissemination (Normanno et al. 2007). The ability of this bacterium to produce iron acquisition factors (siderophores), such as staphyloferrins A and B, staphylobactin and aureochelin, is also likely important to its pathogenesis (Dale et al. 2004; Oogai et al. 2011). Furthermore, the ability of *S. aureus* to form biofilms and colonize medical devices is regarded as an important virulence determinant in the pathogenesis of this bacterium.

Biofilm is a community of microorganisms attached to abiotic or biotic surfaces and embedded in a protective extracellular polymeric matrix (Donlan 2002). The biofilms are formed on abiotic surfaces through multiple steps, including the adhesion of planktonic cells, maturation, and dispersion of attached cells. Sessile *S. aureus* cells are particularly problematic and their physiology differ distinctly from that of planktonic ones. In fact, sessile cells are much more resistant to the host immune response, antibiotics, biocides and hydrodynamic shear force (Lewis 2001; Garrett et al. 2008). The bacterial adhesion to a surface constitutes the first and the essential step of the biofilm formation (Abdallah et al. 2014a). It has been reported that the physicochemical properties of bacterial and abiotic surfaces, such as the hydrophobicity, the electrostatic charge, and the electron donor/acceptor characters, play a key role in the bacterial attachment to abiotic surfaces (Abdallah et al. 2014a). However, another study has underlined that the physicochemical properties have only a minor role and the correlation between the surface properties and the bacterial adhesion were poor (Teixeira et al. 2008). The bacterial detachment is a main part of the biofilm life cycle (Wilson et al. 2004). The phenomenon is involved in the dissemination of infection and contamination in the healthcare and food settings (Nickel et al. 1994; Poulsen 1999). Moreover, Fux et al. (2004) reported that the mechanical biofilm detachment by flushing a colonized catheter provokes sepsis. The erosion of biofilm also results spontaneously, either in the detachment of single cells or clumps of thousands bacteria which contaminate and colonize other surfaces. Thus it is of importance to understand *S. aureus* phenotype changes related to bacterial growth under planktonic and biofilm states. Such investigations might yield important information regarding the virulence and the pathogenicity required for certain acquired human infections.

The purpose of the current work is to investigate the impact of *S. aureus* growth conditions on the physicochemical properties of the biofilm-detached-and the planktonic-cells and on their ability to adhere to the stainless steel (SS) and to the polycarbonate (PC). The planktonic and the biofilm cells were recovered from cultures incubated at different growth temperatures and ages commonly encountered in the medical environments. This work also investigated the effect of these growth conditions on the expression of some virulence factors, involved in the pathogenesis of *S. aureus,* and the cytotoxicity against HeLa cells.

## Materials and methods

### Bacterial strains and culture conditions

The bacterial strain used in this study was *Staphylococcus aureus* CIP 4.83. The strain was stored at − 80 °C in Tryptic Soy Broth (TSB; Biokar Diagnostics, Pantin, France) containing 40% (v/v) of glycerol. Pre-cultures were done by inoculating 100 µl from frozen stock tubes into 5 ml of TSB and then incubated at 20, 30 or 37 °C. The 30 and 37 °C pre-cultures were incubated for 24 h, whereas that of 20 °C was incubated for 48 h. The main cultures were done in 500-ml sterile flasks containing 50 ml of TSB. The cultures of 20, 30 and 37 °C were prepared by inoculating 10^4^ CFU/ml from the 20, 30 and 37 °C pre-culture tubes, respectively. The cultures were then incubated under shaking (160 rpm) at 20, 30 or 37 °C. The cultures were stopped at the late exponential phase.

### Coupons preparation

The SS (304L, Equinox, Willems, France) and PC (Plexilux, Vaux-le-Pénil, France) slides were soaked in ethanol 95° (Fluka, Sigma-Aldrich, Saint-Quentin-Fallavier, France) for an overnight and then rinsed twice with distilled water. Then the slides were soaked in 500 ml of DDM ECO detergent (1%) for 15 min at room temperature (20 °C) under agitation (ANIOS, Villeneuve d’Ascq, France). Slides were thoroughly rinsed five times, for one min under agitation, in 500 ml of distilled water and three times in ultrapure water (Milli-Q^®^ Academic, Millipore, Guyancourt, France) at 20 °C to eliminate detergent residues. SS slides were air-dried and sterilized by autoclaving at 121 °C for 20 min. PC slides were sterilized in the ethanol 95° for 15 min.

### Cell suspension preparation

*Staphylococcus aureus* cells, grown at 20, 30 and 37 °C, were harvested by centrifuging cultures at 5000 g for 5 min at 20 °C. Bacteria were washed twice with 20 ml of 100 mM Potassium Phosphate Buffer (PPB; pH 7) and finally resuspended in 20 ml of PPB. In order to disperse cells, bacterial suspensions were subjected to a sonication at 37 kHz for 5 min at 20 °C (Elmasonic S60H, Elma^®^). The bacterial suspensions at 10^8^ CFU/ml were then prepared by adjusting the optical density to OD_620 nm_ = 0.110 ± 0.005 using a Jenway 6320D UV/visible light spectrophotometer. Standardized cell suspensions (10^8^ CFU/ml) were diluted tenfold for the biofilm formation and the bacterial adhesion assays (10^7^ CFU/ml).

### Biofilm formation assays

Sterile coupons (90 × 90 × 1 mm) were placed in the horizontal position in cell culture dishes (140 mm in diameter). The upper face of slides was covered by 12 ml of 20, 30 and 37 °C cell suspensions (10^7^ CFU/ml) and incubated at 20 °C for 1 h to allow bacterial adhesion. Thereafter, the 12 ml were removed and slides were gently rinsed twice with 12 ml of PPB to remove loosely attached cells. The upper face of slides was covered by 12 ml of TSB and the biofilm formation was started by incubating slides, at the same temperature of bacterial-cell-cultures (20, 30 or 37 °C), for an incubation duration of 24 or 48 h. For the biofilm grown for 48 h, the culture medium was changed after 24 h of biofilm growth, except for DNase, cell cytotoxicity, and siderophore quantification assays where the culture medium was not changed. After 24 and 48 h, supernatants were removed and used for the DNase, the cell cytotoxicity, and siderophore quantification assays. The slides were rinsed twice with 12 ml of PPB in order to remove loosely attached cells. Attached cells were then recovered into 10 ml of PPB by surface scraping. Attached cells were harvested by centrifuging suspensions at 5000 g for 5 min at 20 °C and then washed once with 20 ml of PPB. In order to remove the biofilm matrix, attached cells were resuspended in 20 ml of PPB and suspensions were sonicated at 37 kHz for 5 min at 20 °C. Finally, the attached cells were recovered in 20 ml of PPB. The bacterial suspensions were adjusted to a cell concentration of 10^7^ CFU/ml for the bacterial adhesion assays.

### Adhesion assays

The adhesion of planktonic cells was performed on both SS and PC discs (41 × 1 mm). The adhesion of bacteria detached from biofilms grown on SS and PC was performed respectively on sterile SS and PC using the *NEC Biofilm system* (Abdallah et al. 2015). Sterile coupons of SS and PC were placed in the horizontal position in sterile *NEC Biofilm system*. The upper face of each slide was covered with 3 ml of corresponding-cell-suspensions (10^7^ CFU/ml) and statically incubated at 20 °C for 60 min to allow bacterial adhesion. After 1 h, the slides were removed using sterile forceps and rinsed twice by gently dipping into 30 ml of PPB to remove excess liquid droplets and loosely attached cells. Cells were then stained for 15 min in the dark using Acridine Orange 0.01% (w/v) (Sigma Aldrich, Saint-Quentin Fallavier, France) and then rinsed once by gently dipping in 30 ml of ultrapure water. The attached cells were quantified using epifluorescence microscope (Nikon Optiphot-2 EFD3). A total of 30 fields per coupon was scanned and the stained cells were enumerated. The adhesion rates were presented as a number of bacteria per microscopic field. The results present the average of three independent experiments and in each experiment, two slides were studied.

### Microbial adhesion to solvents (MATS)

The hydrophobicity and the electron donor (basic) or acceptor (acidic) properties of planktonic and biofilm-detached *S. aureus* were determined using the MATS method as described by Bellon-Fontaine et al. (1996). This method is based on the comparison of bacterial affinity to four solvents (Sigma Aldrich, Saint-Quentin Fallavier, France) with different physicochemical properties. The following pairs of solvents were used: chloroform (electron acceptor solvent)/hexadecane (a nonpolar solvent); ethyl acetate (an electron donor solvent)/decane (a nonpolar solvent). Due to the similar Lifshitz–van der Waals components of the surface tension in each pair of solvents, differences between the affinities to solvents would indicate the electron donor and electron acceptor characters of the bacterial surfaces. The affinity of cells to hexadecane was used as a measure of cell surface hydrophobicity.

Experimentally, bacterial suspensions of 10^8^ CFU/ml were prepared in PPB by adjusting the optical density to OD_400 nm_ = 0.8 (A_0_). Then 2.4 ml of each bacterial suspension were added to 0.4 ml of each solvent and then vortexed for 90 s. The mixture was allowed to stand for 30 min to ensure the complete separation of the two phases. Then the optical density of the aqueous phase (A_1_) was measured at 400 nm using a Jenway 6320D UV/visible light spectrophotometer. The affinity of cells to each solvent was subsequently calculated using the following equation: Affinity % = [1 − (A_1_/A_0_)] × 100. The results represent the average of three independent experiments.

### Measurement of zeta potential

The electrostatic properties of *S. aureus* were determined by measuring the zeta potential (ZP) which is derived from the electrophoretic mobility, using the Helmotz–Smoluchowski equation (Bayoudh et al. 2009). The electrophoretic mobility of bacteria cells suspended in PPB was measured using a laser Zeta Compact zetameter (CAD Instruments, Les Essarts-le-Roi, France), by tracking bacteria with a coupled device camera, under an electric field of 80 V. Each bacterial suspension was diluted in PPB to obtain about 70 bacteria per reading. A 1 mM of the KNO_3_ solution was used as the electrolyte and KOH (1 mM) and HNO_3_ (1 mM) were used to adjust the pH to 7.2 (Sigma-Aldrich, Saint-Quentin-Fallavier, France). For each sample, the ZP measurements were repeated five times. Each experiment was performed in duplicate by using two independent cultures.

### Cytotoxicity assay

Supernatants were recovered from biofilms grown on SS and PC, and planktonic cultures, after 24 and 48 h of incubation. Supernatants of planktonic and biofilm cultures, grown at 20, 30 and 37 °C for 24 and 48 h, were collected and the pH was adjusted to 7.2 ± 0.05 using 1 M hydrochloric acid (HCl) (Sigma-Aldrich, Saint-Quentin-Fallavier, France). Next, supernatants were filtered through sterile 0.2 μm Millipore filters. Both planktonic and sessile *S. aureus* supernatants were diluted after being adjusted to similar cell densities based on optical density (620 nm) measurements. The HeLa cell line, derived from cervical carcinoma from a 31-year-old female (ATCC^®^ CCL-2™, ECACC), were cultured and maintained in Dulbecco’s Modified Eagle’s medium (DMEM, Gibco^®^, Thermo Fisher Scientific, Illkirch, France) supplemented with 10% Fetal Bovine Serum (FBS, Gibco^®^) and 1% penicillin–streptomycin (Gibco^®^) in a humidified incubator at 37 °C and 5% CO_2_. Cells were seeded at a cell density of 10^4^ cells/well in a 96-well plate and grown for 48 h before assay. For cytotoxicity assay, the culture medium was replaced with 100 µl of 10% FBS or TSB (pH 7.2) for the negative control or with 100 µl of *S. aureus* culture supernatants. After 3 h of contact, the mixture was aspirated and cells were washed with Phosphate Buffered Saline (PBS, pH 7.4, ThermoFisher Scientific, Illkirch, France). The cell viability was evaluated using Cell Counting Kit-8 (CCK-8, Sigma-Aldrich, Saint-Quentin-Fallavier, France) assay. Briefly, 10 μl of the CCK-8 solution were added to each well containing 100 µl of DMEM with 10% FBS and the plate were incubated for 1 h in the humidified incubator. The absorbance of each well at 450 nm was measured using a microplate reader (PHERA star FS, BMG LABTECH GmbH, Germany). The mean absorbance value of cells non-treated with supernatants was taken as 100% cellular viability. The results represent the average of three independent experiments and each experiment was done in triplicate.

### Deoxyribonuclease (DNase) activity assay

Bacterial supernatants were collected as described above. Enzyme production was tested on DNA agar (Thermo Fisher Scientific, Illkirch, France) by the deposition of 100 µl of each supernatant in 6 mm diameter well. Supernatant volume was allowed to diffuse for 2 h at 4 °C. The plates were incubated at 37 °C overnight. After incubation, wells were flooded with 1 M HCl. DNase production was identified by a halo zone of clearance (DNA degradation) around the supernatant deposition well. The halo zone diameters correlated with the DNase activity in the corresponding supernatant. The results represent the average of three independent experiments and each experiment was done in duplicate.

### Quantitative spectrophotometric assay for siderophore production

The siderophore quantification of *S. aureus*-culture-supernatants is based on Chrome Azurol Sulphonate (CAS assay) according to Schwyn and Neilands (1987). All reagents were purchased from Fluka Sigma-Aldrich (Saint-Quentin-Fallavier, France). Briefly, in order to prepare the CAS assay solution, 6 ml of 10 mM hexadecyltrimethylammonium bromide, 1.5 ml of iron (III) solution (1 mM FeCl_3_6H_2_O, 10 mM HCl), 7.5 ml of 2 mM aqueous CAS solution and 20 ml of 2.5 mM piperazine buffer in H_2_O (pH 5) were mixed in a 100-ml volumetric flask which was then filled with water to afford 100 ml of CAS assay solution. Then, 100 mg of 5-sulfosalicylic were added to the CAS assay solution and stored in the dark.

In order to quantify the siderophores, 0.5 ml of the culture supernatant was mixed with 0.5 ml from the prepared CAS assay solution. After 1 h of incubation at 20 °C, the absorbance (A_630 nm_) is measured by a Jenway 6320D UV/visible light spectrophotometer. The CAS-iron complex color changes from dark blue to orange after the iron chelation by siderophores. The TSB was used as the blank (reference sample). The percentage of siderophore units was estimated as the proportion of CAS color shift using the formula [(Ar − As)/(Ar)] × 100, where Ar is the A_630 nm_ of the reference sample (TSB + CAS assay solution + shuttle solution) and As is the A_630 nm_ of the sample (supernatant + CAS assay solution + shuttle solution).

### Statistics

The results are presented as mean values and their standard error of the mean. Data analysis was performed using Sigma Plot 11.0 (Systat Software Inc.), using one-way ANOVA (Tukey’s method) to determine the significance of differences. Results were considered significant at a *P* value of < 0.05.

## Results

### Effect of growth conditions on the zeta potential of biofilm-detached and planktonic *S. aureus* cells

**Fig. 1:**
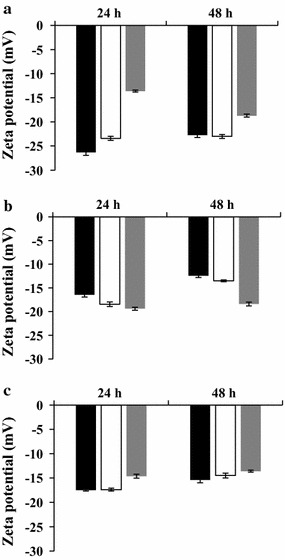
Zeta potential of planktonic and biofilm detached *Staphylococcus aureus* cells. Cell cultures were grown at 20 °C (black square), 30 °C (white square) and 37 °C (grey square), for 24 and 48 h. Planktonic cells (**a**), cells detached from biofilm grown on polycarbonate (**b**), cells detached from biofilms grown on stainless steel (**c**)

This investigation aimed to study the electronegativity of planktonic and biofilm-detached cells in response to different bacterial growth temperatures (20, 30 and 37 °C) and incubation durations (24 and 48 h). For the biofilm formation, two abiotic surfaces, the SS and the PC were used. Figure 1 presents the zeta potential (ZP) values of bacterial surfaces as a function of *S. aureus* growth conditions. The results indicated that *S. aureus* cells were negatively charged, with negative ZP values, whatever the growth conditions (Fig. 1).

Figure 1a showed that the growth temperature and the incubation duration had a significant effect on the ZP of planktonic cells (*P* < 0.05). The increase of growth temperature from 20 to 37 °C significantly increased the ZP of the 24 h planktonic cells from − 26.3 to − 13.6 mV (*P* < 0.05) and the ZP of 48 h planktonic cells from − 22.7 to − 18.7 mV (*P* < 0.05) (Fig. 1a). When cells were grown at 20 °C, the results underlined that the increase of incubation time from 24 to 48 h increased by 1.2-fold the ZP of planktonic cells (*P* < 0.05). However, the increase of the incubation duration of 37 °C planktonic cultures from 24 to 48 h significantly decreased by 1.4-fold the ZP of planktonic cells (*P* < 0.05).

Furthermore, the results showed in Fig. 1 indicated that planktonic cells were significantly more negatively charged than their biofilm-detached counterparts whatever the studied conditions (*P* < 0.05), except for planktonic cells grown at 37 °C for 24 h where the electronegativity of planktonic cell surfaces was lower than that of 24 h biofilm-detached cells (*P* < 0.05). In addition, our findings underlined that the abiotic surface type had a significant effect on the electronegativity of the biofilm-detached cells (Fig. 1b, c). When the growth temperature increased from 20 to 37 °C, the ZP of 24 h and 48 h Polycarbonate-Biofilm-Detached-Cells (PCBDCs) significantly decreased from − 16.4 to − 19.4 mV and from − 12.4 to − 18.4 mV, respectively (*P* < 0.05) (Fig. 1b). The increase of incubation duration from 24 to 48 h had only a significant effect on the electronegativity of PCBDCs grown at 20 and 30 °C. The increase of incubation duration of the biofilm cultures from 24 to 48 h increased by 1.4-fold the ZP of 20 and 30 °C PCBDCs (*P* < 0.05) (Fig. 1b). The stainless steel-biofilm-detached-cells (SSBDCs) showed an opposite electronegativity trend regarding the effect of growth temperature. The Fig. 1c showed that the increase of the biofilm growth temperature from 20 to 37 °C significantly increased the ZP of SSBDCs by 1.2-fold (*P* < 0.05) whatever the incubation durations (Fig. 1c).

### Effect of growth conditions on the cell surface hydrophobicity and electron donor/acceptor characters of biofilm-detached and planktonic *S. aureus* cells

**Fig. 2:**
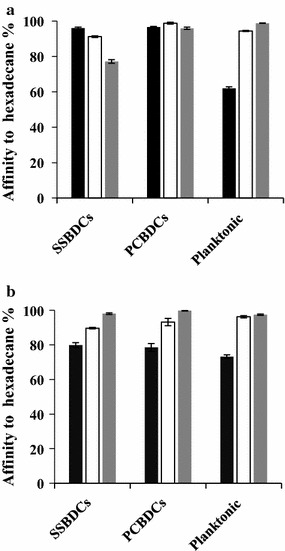
Affinity of planktonic and biofilm detached *Staphylococcus aureus* cells to hexadecane. Cells grown, at 20 °C (black square), 30 °C (white square) and 37 °C (grey square). SSBDCs represents the stainless steel-biofilm-detached-cells. PCBDCs represents the polycarbonate-biofilm-detached-cells. Cells grown during 24 h (**a**) and 48 h (**b**)

This study investigated the physicochemical surface properties of planktonic and biofilm-detached *S. aureus* cells, using the microbial adhesion to solvents (MATS), in response to different incubation durations (24 and 48 h), growth temperatures (20, 30 and 37 °C) and abiotic surfaces (SS and PC). The results related to the hydrophobicity (affinity to hexadecane) and the acceptor/donor character of planktonic and biofilm-detached *S. aureus* cells are shown in Fig. 2 and Table 1.

**Table 1:** Electron donor/acceptor character of biofilm-detached and planktonic *Staphylococcus aureus* cells, grown at 20, 30 and 37 °C, during 24 and 48 h

	T °C^c^	Electron donor^a^			Electron acceptor^b^		
		SSBDCs^d^	PCBDCs^e^	Planktonic	SSBDCs	PCBDCs	Planktonic
24 h	20	1.9 ± 0.1	2.2 ± 0.5	31.2 ± 1.1	17.4 ± 0.5	− 49.6 ± 3.2	3.9 ± 0.6
	30	7.8 ± 0.6	0.9 ± 0.7	4.8 ± 0.5	− 1.5 ± 0.2	− 19.5 ± 2.4	− 31.8 ± 0.2
	37	21.6 ± 0.6	4 ± 0.6	0.7 ± 0.1	− 22.3 ± 0.9	− 17.9 ± 0.7	− 63.6 ± 0.2
48 h	20	18.6 ± 1.6	18.6 ± 2.2	25.2 ± 0.5	7.2 ± 1.3	− 23.5 ± 1.8	− 70.3 ± 2.8
	30	9.8 ± 0.2	6.2 ± 1.8	3.7 ± 0.7	7.1 ± 0.3	− 22.6 ± 2.7	− 38.5 ± 2.6
	37	1.3 ± 0.3	− 1 ± 0.2	0.6 ± 0.1	− 17.7 ± 0.2	− 14.3 ± 3.6	− 25.4 ± 1.7

^a^The differences between the chloroform and hexadecane affinities of cells suspended in 100 mM PPB (pH 7) presents the electron donor character

^b^The differences between the ethyl acetate and decane affinities of cells suspended in 100 mM PPB (pH 7) presents the electron acceptor character

^c^T °C represents the growth temperature

^d^SSBDCs represents the stainless steel-biofilm-detached-cells

^e^PCBDCs represents the polycarbonate-biofilm-detached-cells

The results underlined that the increase of growth temperature of *S. aureus* significantly increased the hydrophobic character of planktonic cell surfaces (*P* < 0.05) (Fig. 2a, b). When cells were grown at 20 °C, the increase of the incubation duration from 24 to 48 h significantly increased the affinity of planktonic cells to hexadecane from 61.9 to 73.2% (*P* < 0.05) (Fig. 2a, b). However, the surface hydrophobicity of planktonic cells grown at 30 and 37 °C was not influenced by the increase of the incubation duration of *S. aureus* cultures (*P* > 0.05). Table 1 showed that planktonic cells have low relative electron acceptor character whatever the growth conditions. However, the electron donor character of planktonic cells grown for 24 h decreased from 31.2 to 0.7 with the increase of growth temperature from 20 to 37 °C. Similar results were observed for planktonic cells grown for 48 h (Table 1). Our findings also showed that, in addition to the incubation duration and the growth temperature, the surface type, had a significant effect on the hydrophobicity as well as the acceptor/donor character of *S. aureus* biofilms-detached cells (Fig. 2a, b). After an incubation duration of 24 h, the surface hydrophobicity of SSBDCs decreased with the increase of the biofilm growth temperature. The affinity of 24 h SSBDCs to hexadecane decreased from 96 to 77% when the biofilm growth temperature increased from 20 to 37 °C (*P* < 0.05) (Fig. 2a). However, an opposite profile was observed for cells recovered from biofilms grown on the SS for 48 h. The affinity of 48 h SSBDCs to hexadecane increased from 80 to 98% when the biofilm growth temperature increased from 20 to 37 °C (Fig. 2b). The affinity of 48 h PCBDCs to the hexadecane increased from 78 to 99% (*P* < 0.05) when the biofilm growth temperature increased from 20 to 37 °C (Fig. 2b). Furthermore, the results showed that the electron donor characters of 24 h SSBDCs increased from 1.9 to 21.6 with the increase of the biofilm growth temperature from 20 to 37 °C. The electron donor character of 24 h PCBDCs increased from 2.2 to 4 when the growth temperature increased from 20 to 37 °C (Table 1). After 48 h of incubation, the electron donor character of SSBDCs and PCBDCs decreased from 18.6 to 1.3 when the growth temperature of biofilms increased from 20 to 37 °C whatever the surface type of the biofilm formation (Table 1). Table 1 also showed that the increase of biofilm growth temperature from 20 to 37 °C significantly decreased the electron acceptor character of 24 h and 48 h SSBDCs from 17.4 to − 22.3 and from 7.2 to − 17.7, respectively (Table 1). The results of Table 1 also showed that PCBDCs presented low relative electron acceptor character whatever the growth conditions.

When cells were grown at 20 °C, the result showed that the electron donor characters of 24 h and 48 planktonic cells were 16 and 1.3-fold higher than those of 24 and 48 h biofilm-detached cells, respectively (Table 1). However, the electron donor character of SSBDCs, grown at 30 and 37 °C, was twofold higher than that of their planktonic counterparts whatever the biofilm incubation duration, except for the 24 h SSBDCs grown at 37 °C where the electron donor character was of 30-fold higher. The results also showed that the electron donor characters of 30 and 37 °C SSDBCs were significantly higher than that of their PCBDCs counterparts whatever the biofilm incubation duration.

### Effect of growth conditions on the adhesion of biofilm-detached and planktonic *S. aureus* cells to stainless steel and polycarbonate

This investigation aimed to study the effect of the *S. aureus* growth conditions on the adhesion behavior of planktonic *S. aureus* cells on SS and PC. The adhesion assays have been done using planktonic cells recovered from cultures grown under different growth temperatures (20, 30 and 37 °C) and durations (24 and 48 h).

**Fig. 3:**
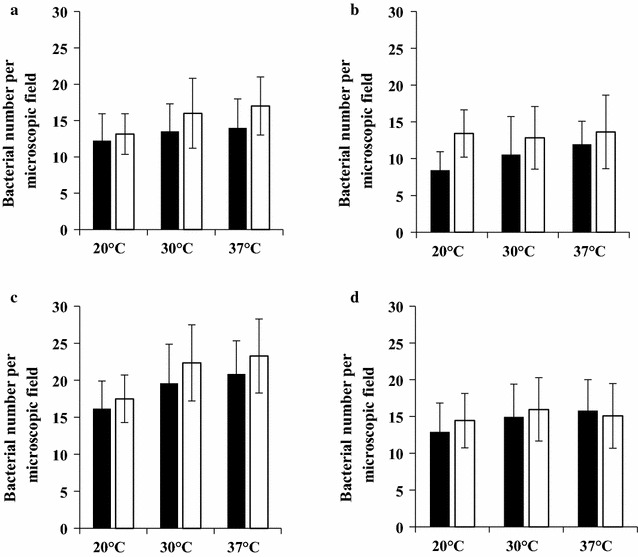
Adhesion of planktonic and biofilm-detached *Staphylococcus aureus* cells on stainless steel and polycarbonate. Cell cultures were grown at 20, 30 and 37 °C, during 24 h (black square) and 48 h (white square). Planktonic cells adhesion on stainless steel (**a**) and polycarbonate (**b**). Adhesion of stainless steel-biofilm-detached-cells on stainless steel 24 (**c**) and polycarbonate-biofilm-detached-cells on polycarbonate (**d**)

Our results showed that the increase of the growth temperature of *S. aureus* from 20 to 37 °C slightly increased by 1.2-fold the adhesion of planktonic cells on the SS whatever the incubation duration (*P* > 0.05) (Fig. 3a). The adhesion experiments performed on the PC showed that the adhesion rate of 24 h planktonic cells increased by 1.2-fold when the growth temperature of *S. aureus* increased from 20 to 37 °C (*P* > 0.05) (Fig. 3b). Figure 3a, b showed that the adhesion rate of 24 and 48 h planktonic cells was respectively 1.4- (*P* < 0.05) and 1.2-fold (*P* > 0.05) higher on the SS than on the PC whatever the growth temperature, except for the planktonic *S. aureus* cells grown for 48 h at 20 °C where the adhesion rates were similar on both studied abiotic surfaces.

This study also investigated the adhesion behaviour of biofilm-detached cells, recovered from biofilms grown under different incubation temperatures (20, 30 and 37 °C), durations (24 and 48 h) and surface types (SS and PC), on the SS and PC. For this study, the adhesion of SSBDCs and PCBDCs was investigated respectively on the SS and the PC.

The results underlined that the abiotic surface type and the temperature of the biofilm formation had an effect on the adhesion behavior of SSBDCs on the SS. Figure 3c showed that the increase of the biofilm growth temperature from 20 to 37 °C increased by 1.3-fold the adhesion rate of 24 and 48 h SSBDCs on theSS (Fig. 3c). However, the Fig. 3d showed that neither the time nor the temperature of biofilm growth had a significant effect on the adhesion rate of PCBDCs on the PC (*P* > 0.05). Furthermore, our data showed that the adhesion rate of SSDBCs on the SS was 1.3-fold higher than the adhesion rate of their PCBDCs counterparts on the PC whatever the studied conditions (Fig. 3c, d). Moreover, Fig. 3a, c showed that the bacterial adhesion rate of SSBDCs on the SS was 1.4-fold higher than the adhesion rate of their planktonic counterparts on the same surface whatever the growth temperature and incubation durations (*P* < 0.05). The adhesion rate of 24 h PCBDCs on the PC was 1.5-fold higher than that of 24 h planktonic cells on the same surface whatever the growth temperature (*P* < 0.05). However, the adhesion rates of 48 h PCBDCs on the PC was similar to that of 48 h planktonic cells on the same surface whatever the growth temperature (*P* > 0.05) (Fig. 3b, d).

### Effect of growth conditions on the production of DNase by *S. aureus* biofilm and planktonic cultures

The assessment of the nuclease activity was realized on supernatants recovered from planktonic cultures and biofilm grown on SS and PC at different growth temperatures (20, 30 and 37 °C) and incubation durations (24 and 48 h). The TSB has been used as a negative control and the results showed that it had no DNase activity (data not shown).

**Fig. 4:**
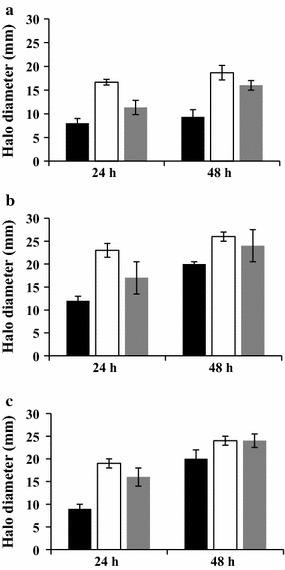
DNase activity in supernatants recovered from *Staphylococcus aureus* planktonic and biofilm cultures. Cell cultures were grown at 20 °C (black square), 30 °C (white square) and 37 °C (grey square), during 24 and 48 h. Planktonic cultures (**a**), biofilms grown on polycarbonate (**b**) and biofilms grown on stainless steel (**c**)

The results showed that the DNase activity of the planktonic-culture-supernatants (PCSs) seems to be dependent on the temperature and the incubation duration of growth. The increase of the growth temperature from 20 to 30 °C significantly (*P* < 0.05) rose the DNase activity of PCSs by twofold whatever the incubation duration (Fig. 4a). When the incubation temperature increased from 20 to 37 °C, the DNase activity of 24 and 48 h PCSs increased respectively by 1.2- and 1.8-fold (*P* > 0.05) (Fig. 4a). The results also showed that the increase of the incubation duration of planktonic cultures from 24 to 48 h significantly increased the DNase activity of 30 and 37 °C PCSs respectively by 1.2- and 1.6-fold (*P* < 0.05) (Fig. 4a).

Our results also showed that the abiotic surface type had a significant effect on the DNase production by sessile *S. aureus* cells and this effect is dependent on the duration and the temperature of biofilm growth. When biofilm growth temperature increased from 20 to 37 °C, the DNase activity of supernatants recovered from 24 h and 48 h SS-biofilms increased respectively by 1.9- and 1.2-fold (*P* < 0.05) (Fig. 4c). The increase of the PC biofilm incubation temperature from 20 to 37 °C increased the DNase activity of 24 and 48 h biofilm-cultures-supernatants (BCSs) respectively by 1.4- and 1.2-fold (Fig. 4b). Furthermore, the results showed that the increase of the incubation duration from 24 to 48 h increased the DNase activity of 20, 30 and 37 °C BCSs respectively by 1.6, 1.1 and 1.4-fold when the biofilms were grown on the PC (*P* < 0.05) and respectively by 2.2, 1.2 and 1.5-fold when the biofilms were grown on the SS (*P* < 0.05) (Fig. 4c). Furthermore, our data underlined that BCSs of *S. aureus* seem to have higher DNase activity than that of PCSs whatever the studied conditions (*P* < 0.05) (Fig. 4a–c).

### Effect of growth conditions on the cytotoxicity of *S. aureus* biofilm and planktonic cultures

**Fig. 5:**
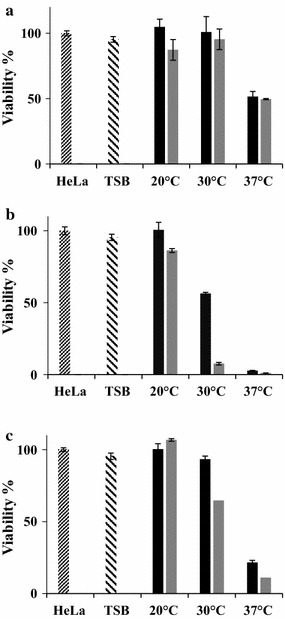
Cytotoxicity of *Staphylococcus aureus* supernatants recovered from planktonic and biofilm cultures. Cell cultures were grown at 20, 30 and 37 °C, during 24 h (black square) and 48 h (grey square). Planktonic cells (**a**), biofilms grown on polycarbonate (**b**) and biofilms grown on stainless steel (**c**)

The planktonic and biofilm culture supernatants, used for the DNase analysis, have been used to test their cytotoxic effects against HeLa cells. This study willed to evaluate the supernatant cytotoxicity of *S. aureus* cells as a function of their growth conditions. The viability of HeLa cells, after an incubation of 3 h with appropriate supernatants, is shown in Fig. 5. The TSB has been used as a negative control. The results showed that TSB, used as a negative control, slightly reduced the viability of HeLa cells by 5% whatever the studied conditions (Fig. 5a–c).

Our findings also underlined that planktonic and biofilm-culture supernatants had a significant effect on HeLa cells viability (*P* < 0.05) and this effect seems dependent on the temperature and the incubation duration of planktonic cultures. The results showed that the PCSs did not affect the HeLa cell viability when cultures are incubated at 20 and 30 °C for 24 and 48 h (Fig. 5a). However, the 37 °C PCSs reduced by twofold (*P* < 0.05) the HeLa cell viability whatever the incubation duration of planktonic cultures (Fig. 5a).

Figure 5b, c also showed that the surface type of the biofilm formation had a significant effect on the cytotoxicity of the BCSs (*P* < 0.05). After an incubation time of 24 h, the cytotoxicity of PC and the SS-BCSs significantly decreased the viability of HeLa cells respectively by 36.4- and 4.6-fold when the biofilm growth temperature increased from 20 to 37 °C (*P* < 0.05) (Fig. 5b, c). Similar data were observed for the 48 h BCSs (Fig. 5b, c). At 20 °C, the biofilm supernatants have not shown a significant cytotoxic effect against HeLa cells whatever the studied conditions (Fig. 5a–c). Furthermore, the PC-BCSs seem to be more cytotoxic than their SS and planktonic counterparts (Fig. 5a–c). After 24 h of incubation, the supernatants of 20, 30 and 37 °C PC-biofilms was respectively 1.3-, 1.2- and 3-fold more cytotoxic than that of their SS counterparts (Fig. 5b, c). After 24 h, the cytotoxicity levels of the 20, 30 and 37 °C PC-BCSs was 1.3, 2 and 24-fold higher (*P* < 0.05) than those of 20, 30 and 37 °C planktonic supernatants, respectively (Fig. 5a, b). This trend was more pronounced when comparing the supernatant cytotoxicity of 48 h aged biofilm to the 48 h planktonic ones (*P* < 0.05) (Fig. 5a, b). In fact, the cytotoxicity of the PC and SS culture supernatants were respectively 57 and fivefold higher than that of planktonic cultures (Fig. 5a–c).

### Effect of growth conditions on siderophore production by *S. aureus* biofilm and planktonic cultures

**Fig. 6:**
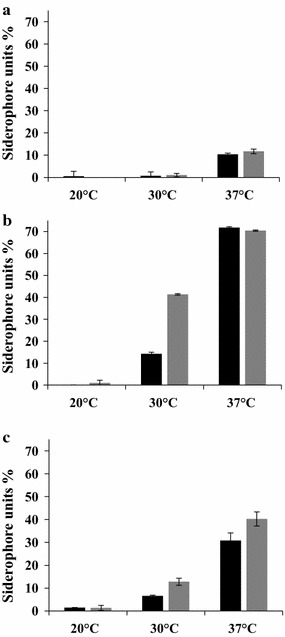
Quantification of siderophore in *Staphylococcus aureu*s culture supernatants. Cell cultures were grown at 20, 30 and 37 °C, during 24 h (black square) and 48 h (grey square). Planktonic cells (**a**), biofilm grown on polycarbonate (**b**), biofilm grown on stainless steel (**c**)

The goal here is to investigate the effect of incubation duration, growth temperature and surface type on the siderophore production by planktonic and biofilm S*. aureus* cells (Fig. 6). The results of Fig. 6a showed no detectable siderophore production when planktonic cells were grown at 20 and 30 °C whatever the incubation durations. However, the planktonic cells grown at 37 °C exhibited 11% of siderophore units whatever the incubation duration of planktonic cultures (Fig. 6a). Our findings also showed that the surface type, the growth temperature, and the incubation duration had a significant effect (*P* < 0.05) on siderophores production by sessile *S. aureus* cells (Fig. 6b, c). When grown on the SS, the increase of the biofilm growth temperature from 20 to 37 °C significantly increased the percentage of siderophores units of 24 h and 48 h biofilm supernatants from 1.4 to 30.8% and from 1.3 to 40.2%, respectively (*P* < 0.05) (Fig. 6c). When *S. aureus* biofilms are grown on the PC, the percentage of produced siderophore units increased from an undetectable level to 71% (*P* < 0.05) when the biofilm growth temperature increased from 20 to 37 °C whatever the incubation duration of the biofilm formation (Fig. 6b). In addition, our data showed that the amount of produced siderophore by sessile cells grown on PC was significantly higher than that of their planktonic and SS counterparts whatever the studied conditions (*P* < 0.05) (Fig. 6a–c).

## Discussion

Bacterial adhesion and biofilm formation have become a serious problem in healthcare and food sectors, and much investigations have been done for better understanding of the processes involved. However, most of studies have focused on the bacterial adhesion of planktonic cells but have not considered the biofilm-detached cells which may be involved in contamination spread. It has been reported that the physiology of planktonic and biofilm-detached cells are deeply different (Stewart and Costerton 2001; Donlan and Costerton 2002). In this context, our study investigated, in particular, the impact of growth conditions on the physicochemical properties of biofilm-detached and planktonic *S. aureus* cells and on their ability to adhere to the SS and PC. Overall, our results showed that the increase of temperature and the incubation duration slightly increased the adhesion of *S. aureus* to the SS and the PC. These results are in agreement with previous studies which highlighted the effect of these parameters on the adhesion of *S. aureus* (Abdallah et al. 2014b), *Listeria monocytogenes* (Gordesli and Abu-Lail 2012) and *Escherichia coli* (Tsuji and Yokoigawa 2012) to different surfaces. Furthermore, our results showed that biofilm-detached cells had a higher adhesion rate than that of their planktonic counterparts. The same trend was reported by Berlanga et al. (2015), who underlined the greater ability of biofilm-detached *Halomonas venusta* cells to colonize new surfaces compared to their planktonic counterparts. By contrast, other studies (Allison et al. 1990), reported that there were no significant differences between the adhesion of biofilm-detached and planktonic *E. coli* cells to abiotic surfaces. Furthermore, we investigated the effect of growth conditions on the surface physicochemical properties of *S. aureus* cells. Overall, we showed that the hydrophobicity of biofilm-detached and planktonic *S. aureus* cells increased when the growth temperature increased from 20 to 37 °C. These findings seem to be consistent with those of Abdallah et al. (2014a), who found that the hydrophobicity of *S. aureus* increased with the increase of the growth temperatures. Therefore, this result may explain the increase of *S. aureus* adhesion onto SS and PC. However, and if we consider, particularly, the results related to the bacterial surface hydrophobicity we could suggest that cell adhesion should be greater on hydrophobic supports such as the PC which is not the case under our experimental conditions. In accordance with a previous study (Abdallah et al. 2014a), our results showed a greater adhesion rate of *S. aureus* cells on SS than on the PC. Such results highlight that the hydrophobic interactions cannot always explain the bacterial adhesion onto abiotic surfaces. It has been reported that the acid–base interactions are the main forces governing the bacterial adhesion to abiotic surfaces (Bos et al. 1999). Our study highlighted the decrease of the electron donor character of 48 h-biofilm-detached cells with the increase of growth temperature. This may result in a decrease of repulsive acid–base interactions between the cells and the abiotic surfaces. Such decrease may, therefore, explain the increase of the bacterial adhesion of 48 h-biofilm-detached cells on the SS. By contrast, our results also showed that the electron donor characters of *S. aureus* did not always explain the differences found in the experimental results. In fact, the increase of electron donor character of 24 h-biofilm-detached-cells, with the increase of growth temperature from 20 to 37 °C, was accompanied by an increase of the bacterial adhesion on both surfaces. Furthermore, we investigated the involvement of electrostatic interactions in the *S. aureus* adhesion to the SS and the PC. Our results showed that the ZP of *S. aureus* cells was negative whatever the studied conditions. Our findings also showed that biofilm-detached cells are less negatively charged than their planktonic counterparts, probably due to the up-regulation of cationic staphylococcal poly-*N*-acetylglucosamine surface polysaccharide (Otto 2008). Therefore, the low relative negative charge of biofilm-detached cells may result in a decrease of repulsive electrostatic forces between cells and negatively charged abiotic surfaces, which may explain their greater adhesion rates on abiotic surfaces as compared to that of their planktonic counterparts. Furthermore, our results showed that the increase of the growth temperature may result in a decrease of repulsive electrostatic interactions, between negatively-charged bacterial cells and abiotic surfaces. Therefore, this may explain the enhanced adhesion of the biofilm-detached *S. aureus* cells onto the SS. By contrast, our data showed that the increase of the growth temperature resulted in a decrease of the zeta potential of the biofilm-detached cells and simultaneously in an increase of the bacterial adhesion to the PC. Hence, we suggest that the electrostatic interactions may not always explain the bacterial adhesion to abiotic surfaces which involves other factors related to the cell envelope in this process (Hori and Matsumoto 2010). This work also investigated the effect of the growth conditions on the pathogenicity and cytotoxicity of the different studied *S. aureus* cultures. The DNase activity of biofilm cultures was greater than that of the planktonic cultures. In addition, the results showed that the DNase activity increased with the increase of the growth temperature and the incubation duration. These results are in disagreement with other studies (Resch et al. 2005; Wang et al. 2011), which underlined that the virulence factor production by planktonic *S. aureus* was greater compared to that of biofilm cultures. However, our results seem in line with those of Coenye et al. (2007), who stated that the sessile *Propionibacterium acnes* cells produced more virulence factors than the planktonic ones and this production increased with the increase of the incubation time. The present findings also appear to be in agreement with those of Mahoney et al. (2010), who underlined that the bacterial virulence regulation is influenced by the growth temperature. Furthermore, our findings showed that BCSs had a higher cytotoxic effect, on HeLa cells, than the PCSs whatever the studied condition. The cytotoxic effect of BCSs and PCSs increased in response to the increase of the temperature and the incubation duration. Taken together, our results may explain the influence of growth conditions on the bacterial metabolism controlling the production of virulence factors (Holler et al. 1998). According to Secor et al. (2011), the different metabolic states in planktonic and biofilm cultures likely have a large impact on the pathogenic effects on human cells. Thus, in our case, the important cytotoxic effect of *S. aureus* BCSs compared to that of PCSs could be related to the presence of higher amounts of virulence factors including exoenzymes such as DNase, which may disturb the biological activity of human cells (Modun and Williams 1999; Pancholi and Chhatwal 2003; Jarosław et al. 2005; Secor et al. 2011). Nevertheless, our results showed that BCSs recovered from biofilms grown on the PC surface were more cytotoxic to HeLa cells than those of biofilm grown on SS. Interestingly, our investigation showed that the siderophore production, which is enhanced under iron-limiting conditions (Vasil and Ochsner 1999; Gaonkar 2015), in the supernatant of biofilm grown on PC were higher than that of biofilm grown on SS. It has been reported that iron and nickel could be released from the SS into solution (Ortiz et al. 2011). Therefore, the limited availability of iron in the medium of biofilm grown on the PC, which is a plastic surface, could enhance the production of siderophores (Gaonkar 2015). In *S. aureus*, the greater production of siderophores correlated with higher virulence and more resistant (Rozalska et al. 1998; Dale et al. 2004). Taken together, our findings and previous studies may explain the greater cytotoxicity and pathogenicity of supernatants recovered from *S. aureus* biofilms grown on the PC when compared to those recovered from biofilms grown on the SS.

In conclusion, this study showed that biofilm-detached-cells are phenotypically distinct from planktonically grown cells. Moreover, our results showed that the bacterial history and the growth conditions affect the adhesion of *S. aureus* to abiotic surfaces by influencing the bacterial surface physicochemical properties. Our investigations also underlined the hazardous characters of biofilm-detached cells which appeared to be abler to adhere to abiotic surfaces than their planktonic counterparts. Such results highlight the importance of considering cell detachment as a serious stage in the process of biofilm development. These results should contribute to more effective management of disinfection strategies, especially by ensuring a rapid removal and killing of cells detached from contaminated surfaces to prevent the persistence and the spread of contamination. However, our findings underlined that the bacterial physicochemical properties cannot always fully explain the bacterial adhesion. An interesting perspective would consist in focusing on the quantification of bacterial adhesion forces using atomic force microscopy in order to extend the knowledge of the mechanisms mediating bacterial adhesion to abiotic surfaces and to develop new strategies for the prevention of the biofilm formation. In addition, our results showed that sessile cells produce higher amounts of different virulence factors which represent a serious threat in case of human infection by *S. aureus.* Interestingly, growth temperatures close to that of the human body increased the cells virulence potential and cytotoxicity to human cells. Moreover, biofilm formed on plastic surfaces, such as PC, showed higher pathogenic risk than those formed on metallic ones, such as SS. Thus, our results highlight that the presence biofilm on plastic indwelling medical devices such as catheters, may increase the risk of severe infections. Our work offers a novel insight into the infectious potential of *S. aureus*, which suggests that a virulent strains may increase their virulence by forming a biofilm and achieve persistent infection in vivo.

## Abbreviations

*S. aureus*: *Staphylococcus aureus*
°C: degree centigrade
h: hour
SS: stainless steel
PC: polycarbonate
TSB: tryptic soy broth
%: percent
µl: microliter
ml: milliliter
mm: millimeter
µm: micrometer
nm: nanometer
T: temperature
CFU: colony-forming unit
min: minutes
PPB: potassium phosphate buffer
mM: millimolar
M: molar
kHz: kilohertz
MATS: microbial adhesion to solvents
ZP: zeta potential
H_2_O: water
KNO_3_: potassium nitrate
KOH: potassium hydroxide
HCL: hydrogenchloride
CO_2_: carbon dioxide
FBS: phosphate buffered saline
DMEM: Dulbecco’s modified eagle’s medium
CCK: cell counting kit
DNA: deoxyribonucleic acid
DNase: deoxyribonuclease
CAS: chrome azurol sulphonate
mV: millivolt
PCBDC: polycarbonate-biofilm-detached-cells
SSBDC: stainless steel-biofilm-detached-cells
PCS: planktonic-culture-supernatants
BCS: biofilm-culture-supernatants
g: gravitational acceleration
