# Correction to: Effect of incubation duration, growth temperature, and abiotic surface type on cell surface properties, adhesion and pathogenicity of biofilm-detached *Staphylococcus aureus* cells

**DOI:** 10.1186/s13568-017-0509-8

**Published:** 2017-12-05

**Authors:** Simon Oussama Khelissa, Charafeddine Jama, Marwan Abdallah, Rabah Boukherroub, Christine Faille, Nour-Eddine Chihib

**Affiliations:** 1 CNRS, ENSCL, UMR 8207-UMET-PSI, Université de Lille1, Avenue Dimitri Mendeleïev, CS 90108, 59652 Villeneuve d’Ascq, France; 2 CNRS, INRA, UMR 8207-UMET-PIHM, Université de Lille1, 369 rue Jules Guesde, CS 20039, 59651 Villeneuve d’Ascq, France; 3 CNRS, IEMN, UMR 8520, Université de Lille1, Cité Scientifique, Avenue Poincaré, 59652 Villeneuve d’Ascq, France

## Correction to: AMB Expr (2017) 7:191 10.1186/s13568-017-0492-0

Following publication of the original article (Khelissa et al. 2017), the authors reported that the legend for Fig. 3 contained an error. Instead of “Adhesion of planktonic and biofilm-detached *Staphylococcus aureus* cells on stainless steel and polycarbonate. Cell cultures were grown at 20, 30 and 37 °C, during 24 h (white square) and 48 h (white square). Planktonic cells adhesion on stainless steel (**a**) and polycarbonate (**b**). Adhesion of stainless steel-biofilm-detached-cells on stainless steel 24 (**c**) and polycarbonate-biofilm-detached-cells on polycarbonate (**d**)”, the legend should read “Adhesion of planktonic and biofilm-detached *Staphylococcus aureus* cells on stainless steel and polycarbonate. Cell cultures were grown at 20, 30 and 37 °C, during 24 h (black square) and 48 h (white square). Planktonic cells adhesion on stainless steel (**a**) and polycarbonate (**b**). Adhesion of stainless steel-biofilm-detached-cells on stainless steel 24 (**c**) and polycarbonate-biofilm-detached-cells on polycarbonate (**d**)”.

The original article has been corrected.
